# The expression level of alpha-synuclein in different neuronal populations is the primary determinant of its prion-like seeding

**DOI:** 10.1038/s41598-020-61757-x

**Published:** 2020-03-17

**Authors:** Josquin Courte, Luc Bousset, Ysander Von Boxberg, Catherine Villard, Ronald Melki, Jean-Michel Peyrin

**Affiliations:** 1 Sorbonne Universités, Faculté des Sciences et Technologie, CNRS UMR 8246, INSERM U1130, Neurosciences Paris Seine, Institut de Biologie Paris Seine, Paris, 75005 France; 2 Laboratory of Neurodegenerative Diseases, Institut François Jacob, MIRCen, CEA-CNRS, Fontenay aux, Roses, 92265 France; 3 Physico-Chimie Curie, Université PSL, CNRS, Institut Pierre-Gilles de Gennes pour la Microfluidique, Paris, France

**Keywords:** Mechanisms of disease, Parkinson's disease

## Abstract

Alpha-synuclein (aSyn)-rich aggregates propagate in neuronal networks and compromise cellular homeostasis leading to synucleinopathies such as Parkinson’s disease. Aggregated aSyn spread follows a conserved spatio-temporal pattern that is not solely dependent on connectivity. Hence, the differential tropism of aSyn-rich aggregates to distinct brain regions, or their ability to amplify within those regions, must contribute to this process. To better understand what underlies aSyn-rich aggregates distribution within the brain, we generated primary neuronal cultures from various brain regions of wild-type mice and mice expressing a reduced level of aSyn, and exposed them to fibrillar aSyn. We then assessed exogenous fibrillar aSyn uptake, endogenous aSyn seeding, and endogenous aSyn physiological expression levels. Despite a similar uptake of exogenous fibrils by neuronal cells from distinct brain regions, the seeded aggregation of endogenous aSyn differed greatly from one neuronal population to another. The different susceptibility of neuronal populations was linked to their aSyn expression level. Our data establish that endogenous aSyn expression level plays a key role in fibrillar aSyn prion-like seeding, supporting that endogenous aSyn expression level participates in selective regional brain vulnerability.

## Introduction

aSyn is abundantly expressed in neurons^1^, and mostly localizes at synapses where it regulates presynaptic vesicles turnover^2^. aSyn is also a constituent of Lewy bodies and neurites^3^, also named Lewy pathology (LP), the hallmarks of a group of neurodegenerative diseases named synucleinopathies that comprise Parkinson’s disease (PD), multiple system atrophy (MSA), and dementia with Lewy bodies (DLB)^4,5^. The protein is phosphorylated at Ser129 (pSyn)^6^ and ubiquitinated^7^ in aSyn-rich pathogenic protein and lipid deposits^8^. The spatio-temporal spread of LP in the brain of PD patients proposed by Braak and co-workers^9,10^ together with the finding that LP affects young healthy neuronal grafts in the brain of PD patients^11,12^, led to hypothesize that aSyn-rich aggregates have prion-like propensity. This led numerous groups to inject aSyn assemblies made *de novo*, or brain homogenates containing LP into the CNS of model animals, demonstrating that those assemblies trigger LP^13–23^ and have prion-like propensity (reviewed in^24^). Importantly, in patients, the spatio-temporal pattern of LP matched the observed symptoms^25,26^.

How aSyn assemblies spread from neuron to neuron and amplify is not yet fully understood. A number of steps have been nonetheless identified and documented. The first step is the uptake of exogenous seeds from the extracellular space by neuronal cells, possibly through receptor-mediated non-conventional endocytosis^27–31^. Following uptake, the seeds have been shown to reach the cytosol by escaping from the endolysosomal compartment^32–34^. Within the cytosol, exogenous aSyn seeds grow by recruiting endogenous monomeric aSyn^35–40^. They are also actively transported anterogradely and retrogradely, within endosomes or naked, and are released in the extracellular milieu^29,41–47^.

LP sequential development in model animals follows interconnected brain regions^14,18,20,21,23^ and suggests trans-neuronal transfer, although it is important to note that the exact role of synaptic elements in mediating propagation is yet to be addressed. Intriguingly, LP spread pattern does not fit the synaptic weights within the sequentially affected regions^48^, suggesting that synaptic connectivity alone does not define the selective vulnerability of different brain regions in distinct synucleinopathies. It is thus of prime importance to determine what factors govern selective tropism and subsequent vulnerability in order to better understand disease progression in the brain of patients. We can reasonably hypothesize that tropism and vulnerability are defined by specific characteristics of the neuronal cells constituting different brain areas and sub-areas. These characteristics are many, and range from cell surface properties, endo- and exocytosis activities, the extracellular matrix, membrane proteins and phospholipid composition of the plasma membrane, to the distinct proteomes of different neuronal cells.

Here we assess the tropism of the aSyn fibrillar polymorph “Fibrils” for primary striatal (Str), cortical (Cx) and hippocampal (Hip) neurons derived from wild-type (WT) and *SNCA*−/− mouse embryos. We show that different neuron types bind and take up fluorescently labelled aSyn Fibrils to similar extents. We demonstrate nonetheless that the extent to which exogenous aSyn Fibrils seed the aggregation of endogenous aSyn is dependent on the origin of neuronal populations. This prompted us to compare expression level and sub-cellular localization of aSyn in different neuronal populations, and exogenous fibrils seeding propensity in those neurons. We report that aSyn expression level and sub-cellular localization differ in neurons of different origins, and that the differences we observe correlate with exogenous aSyn Fibrils seeding propensity within those neurons. To strengthen this finding, we selectively decreased aSyn expression level in Hip neurons by crossbreeding WT and *SNCA*−/− mice to generate *SNCA*+*/−* heterozygous embryos. aSyn expression level in *SNCA*+*/−* Hip neurons was lower than that in WT Hip neurons, as was the seeding propensity of exogenous aSyn Fibrils in those cells. Taken together, our data suggest that the expression level of aSyn is a key element in aSyn prion-like seeding, and might thus strongly impact the pattern of LP development in heterogeneous neural networks.

## Results

### aSyn Fibrils bind to and are taken up by neuronal populations with similar efficiencies

**Figure 1:**
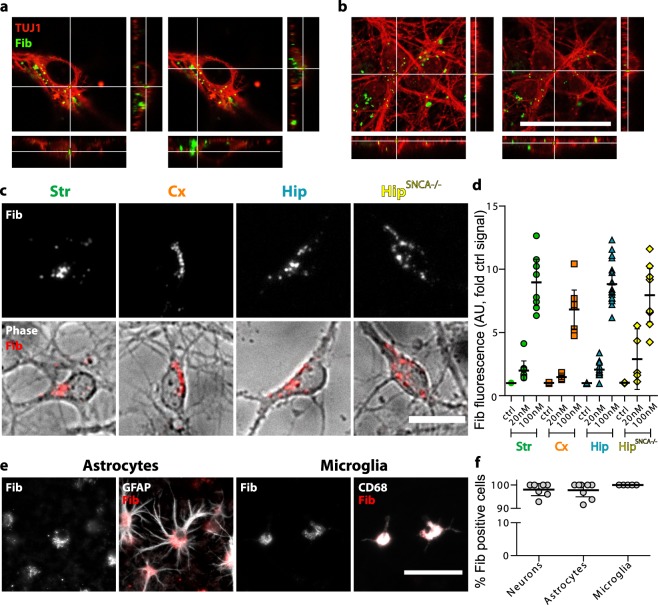
aSyn Fibrils are similarly endocyted in primary neuronal cultures from different brain regions. Primary neuronal cultures from WT or *SNCA*−/− embryos were exposed to 100 nM Fibrils at DIV7 and observed at DIV14. (**a**) Confocal imaging in fixed cells revealed that aSyn Fibrils were present in the neuronal lumen, as evidenced by localization of exogenously added aSyn Fibrils (green) in neuronal somas stained with TUJ1 (red) in Hip neurons and (**b**) in Cx neurons. Scale bar represents 50 µm. (**c**) aSyn Fibrils are endocyted by Str, Cx, Hip and Hip^*SNCA*−/−^ neurons, as evidenced by epifluorescence imaging. aSyn Fibrils fluorescence in red is superimposed to phase contrast images (grey). Scale bar represents 20 µm. (**d**) Quantification of the mean aSyn Fibrils signal in neuronal somas in live cell cultures. 5 to 14 replicates from 2 to 3 individual experiments. Means+*/−* 95% confidence interval (CI95) are shown. (**e**) aSyn Fibrils are taken up by astrocytes and microglia, as evidenced by epifluorescence imaging. Scale bar represents 100 µm. (**f**) Quantification of the percentage of neuronal (MAP2 positive) or glial cells (GFAP and CD68 positive) containing aSyn Fibrils in fixed primary cultures. 5 to 8 replicates from 3 individual experiments. Means +/− CI95 are shown.

The uptake of extracellular seeds into the intracellular space is among the early and key steps of the prion-like aSyn amplification process. In order to assess quantitatively the uptake of aSyn Fibrils by different neuronal populations, we established in microfluidic culture systems primary neuronal cultures of striatal (Str), cortical (Cx) and hippocampal (Hip) cells from wild type (WT) and *SNCA*−/− mouse embryos. These cultures were made up of 35 to 65% of neurons (Supp. Fig. 1a,b), and contained a comparable amount of astrocytes (Supp. Fig. 1c,d). The percentage of inhibitory neurons was consistent with what has been reported *in vivo*, with a majority of Str neurons expressing GAD67, while Cx and Hip neurons were mostly negative for GAD67 (Supp. Fig. 1e,f). We exposed these cultures to Atto550-labeled murine aSyn Fibrils (100 nM, equivalent monomeric aSyn) for 24 hours 7 days after cell plating. The amount of aSyn Fibrils that were taken up was next determined by live epifluorescent microscopy imaging, and Fibrils intracellular localization was assessed by confocal microscopy (Fig. 1a,b). Str, Cx, Hip from WT and *SNCA*−/− mice were all found to efficiently take up aSyn Fibrils (Fig. 1c). The fluorescence levels of Str, Hip and Hip^*SNCA*^−/− neurons cell bodies exposed to 20 or 100 nM of Fibrils were similar. More precisely, the fluorescence intensity increased 2 to 3, and 8 to 9 fold as compared to control unexposed neurons in cultures exposed to 20 or 100 nM of Fibrils, respectively (Fig. 1d).

A significant proportion of aSyn Fibrils was also located in neuronal processes and non-neuronal cells. Notably, Fibrils were also detected in astrocytes and microglia (Fig. 1e), with respectively 97% and 100% of aSyn Fibrils positive cells (Fig. 1f). Thus, Fibrils were efficiently taken up by neuronal and glial cells, and the extent of uptake was similar in neuronal populations derived from distinct brain regions. aSyn expression did not impact Fibrils endocytosis, as evidenced by similar uptake in WT and *SNCA*−/− neurons.

### Differential pSyn accumulation in distinct neuronal populations

Exogenous aSyn fibrils have been shown to seed the aggregation of endogenous aSyn. The recruited aSyn is heavily phosphorylated (pSyn)^6^. We thus quantified the amount of pSyn in Str, Cx and Hip neurons from WT and *SNCA*−/− mice by immuno-staining with anti pSyn antibodies.

**Figure 2:**
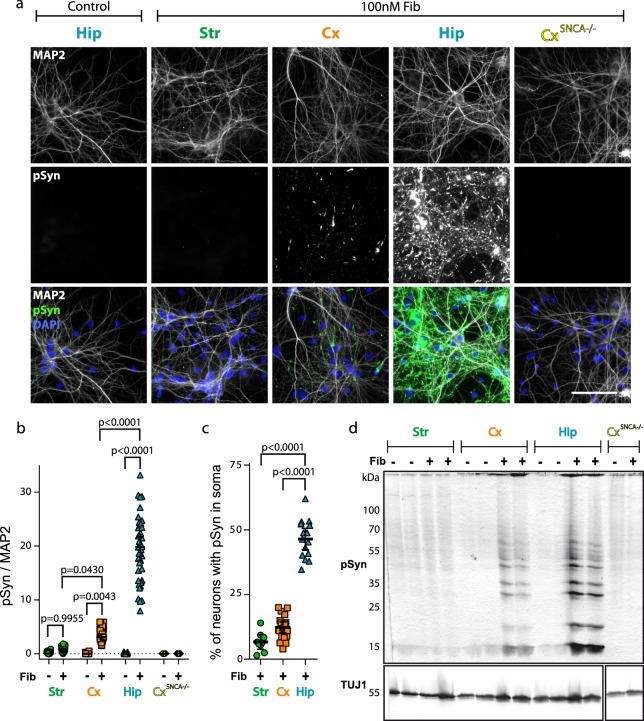
aSyn fibrils seed endogenous aSyn to different extent in primary neuronal cultures from different regions. Primary neuronal cultures from different brain regions of WT or *SNCA*−/− embryos were exposed to 100 nM aSyn Fibrils or to vehicle at DIV7 and observed at DIV21. (**a**) Fixed cells were stained for MAP2 (grey), pSyn (green) and Hoechst (blue) and imaged by epifluorescence microscopy. Scale bar represents 100 µm. (**b**) pSyn area/MAP2 area at fixed thresholds. 5 to 42 replicates from 2 to 11 individual experiments. Two-way ANOVA was performed on experiments means, followed by Tukey’s multiple comparisons test. Adjusted p values are shown. (**c**) Quantification of the percentage of neurons with somatic pSyn. 10 to 20 replicates from 3 to 5 individual experiments. Means +/− CI95 are shown. Brown-Forsythe ANOVA tests and Welch’s ANOVA test were performed, followed by Dunnett’s T3 multiple comparisons test. Adjusted p values are shown. (**d**) Primary neuronal cultures were exposed to 100 nM aSyn Fibrils or to vehicle at DIV7, lysed at DIV14 and the amount of pSyn and TUJ1 quantified by Western blotting. The loading control gel was not loaded in the same order as the gel for pSyn staining in order to reveal high molecular weight pSyn species. Thus, the gel containing loading controls had to be cropped and rearranged. Full length gel is available in Supp. Fig. S9.

Primary Str, Cx and Hip neuronal cells from WT and *SNCA*−/− mice were exposed to aSyn Fibrils (100 nM, equivalent monomeric aSyn) for 7 days, then fixed, immuno-stained and imaged 7 days later. In a separate experiment, a concentration dependent accumulation of pSyn was observed upon exposure of exogenous aSyn fibrils to Hip neurons (Supp. Fig. S2). Interestingly, exogenous aSyn Fibrils and endogenous pSyn aggregates exhibited distinct distribution patterns. Both in glial and neuronal cells, exogenous Fibrils were observed within subcellular compartments, mostly in somata and neurites in neurons, and appeared as puncta whose size (in the micrometre range) is incompatible with the size of individual fibrils (tens of nanometres) (Supp. Fig. S3). Seeded, aggregated pSyn exhibited distinct morphological features, appearing as rods of several micrometres, localizing first in neurites before invading the perinuclear part of the somata (Supp. Fig. S4). Interestingly, the exogenous Fibrils and the endogenous pSyn assemblies only partially co-localize, in agreement with our previous report showing that a fraction of internalized Fibrils escapes the endolysosomal compartment and gains access to the cytoplasm where they seed the aggregation of endogenous aSyn^32^. Fourteen days following exposure to exogenous Fibrils (100 nM), all cells except cortical neurons from *SNCA*−/− embryos (Cx^SNCA^−/−) exhibited to some extent intracellular pSyn staining (Fig. 2a). This demonstrates that pSyn staining corresponds to aggregates made up of endogenous aSyn and not from exogenous assemblies. Strikingly, however, the amount of pSyn greatly differed between Str, Cx and Hip neurons. While Str neurons displayed rare and mostly small pSyn particles, Cx neurons contained numerous ones, which were both fibrillar and punctiform. Hip neurons exhibited the highest number of pSyn particles. Some of these endogenous aggregates reached remarkable sizes (up to 50 µm long) and spanned several neuritic branching points. We quantified the pSyn signal and normalized it by dividing it by the MAP2 signal (Fig. 2b). This pSyn/MAP2 ratio was close to zero in all unexposed cells, and in Cx^SNCA^−/− neurons exposed to Fibrils. Str neurons exhibited very few aggregates, with a 5.8 times lower pSyn/MAP2 ratio than Cx neurons, which themselves had a 5.7 lower ratio than Hip neurons (Fig. 2b). We then quantified the percentage of neurons positive for somatic pSyn assemblies (Fig. 2c). 7% of Str, 11.2% of Cx and 47.3% of Hip neurons exhibited somatic pSyn, suggesting that a subpopulation of neurons within each of the cultured brain regions was more sensitive to aSyn nucleation.

Strikingly, while pSyn accumulation significantly differed between primary cultures of different origins after exposure to exogenous Fibrils, quantification of the percentage of condensed nuclei did not reveal a significant change in cellular death rate in Str, Cx or Hip neurons from WT animals (Supp. Fig. S5).

Pure striatal cultures lead to neurons showing a partially differentiated phenotype compared to their counterparts grown in mixed primary cultures. To ascertain that the differential seeding within each neuronal culture is not due to partial differentiation, we exposed Str neurons grown alone or in contact with Cx^SNCA−/−^ axons to 100 nM Fibrils, based on our previous demonstration using microfluidic culture chips that fully mature Str neurons can be reliably obtained when grown in contact with Cx axon terminals^49^. Str neurons in contact with Cx axons showed an increased susceptibility to exogenous Fibrils seeding. Nonetheless, the amount of pSyn within innervated Str neurons was lower than that in Cx and Hip neurons (Supp. Fig. S6, Fig. 2). Thus, the relatively low Str neuron vulnerability is not substantially altered by innervation-dependent maturation.

Western blot analysis of cell lysates 7 days following exposure to Fibrils yielded results coherent with pSyn immunofluorescence analysis (Fig. 2d). Indeed, while Cx^SNCA−/−^ neurons exposed to Fibrils exhibited no pSyn signal (Fig. 2d, right lanes), we observed a very strong pSyn signal in Hip neurons at molecular weights corresponding to monomeric and multimeric aSyn. The pSyn signal in Cx neurons was lower than that observed for Hip neurons, and the signal in exposed Str neurons was undistinguishable from the one of unexposed cultures. Overall, our observations demonstrate that pSyn accumulation after exposure to Fibrils differs considerably between neurons of different origins in culture.

### Endogenous neuronal aSyn expression level matches exogenous Fibrils-mediated pSyn accumulation

**Figure 3:**
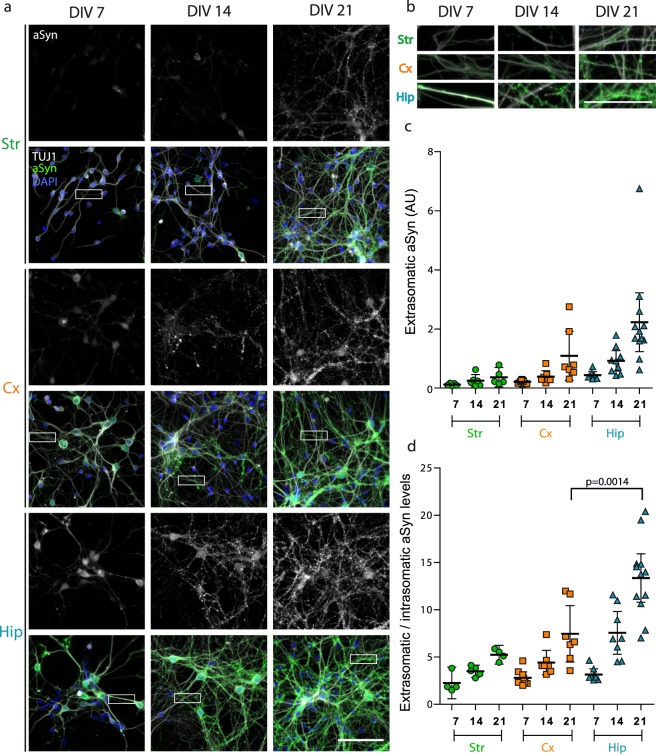
Relationship between aSyn expression and synaptic enrichment and pSyn accumulation in primary neurons. Primary neuronal cultures from different brain regions of WT embryos were fixed at DIV7, 14 or 21. (**a**) Cells were imaged with an epifluorescence microscope after staining for aSyn (green), TUJ1 (grey) and Hoechst (blue). Scale bar represents 100 µm. (**b**) Zoom on neuritic segments of images in (**a**). Scale bar represents 50 µm. (**c**) Quantification of the extra-somatic (synaptic) aSyn signal, normalized by the number of neuronal somas in the imaged field. 5 to 8 replicates from 2 to 3 individual experiments. Means +/− CI95 are shown. (**d**) Ratio of intra-somatic /extra-somatic (synaptic) aSyn. 4 to 12 individual replicates from 2 to 3 individual experiments. Means +/− CI95 are shown. Two-way ANOVA was performed, followed by Tukey’s multiple comparisons test. An adjusted p value is shown.

A partial correlation between aSyn expression levels in specific brain regions and their propensity to accumulate LP has been recently proposed^50^. Brain regions are composed of many different cell types, and SNCA has been reported to be differentially expressed amongst neuronal subpopulations^51–55^. We thus hypothesized that aSyn expression level in the primary neuronal cultures studied here could match their specific vulnerability to exogenous Fibrils-mediated seeding. Str, Cx and Hip neurons were cultured for 7, 14, or 21 days. After fixation, we stained them for aSyn, Hoechst and TUJ1 to assess aSyn expression level and subcellular localization (Fig. 3a). The level of aSyn staining increased from 7 to 21 days of culture in all neuronal cultures. The intensity of aSyn staining differed between neurons, with a stronger signal in Hip compared to Cx neurons, and Cx compared to Str neurons. This difference was conserved over culture time. aSyn progressively acquired synaptic localization during neuronal development (Fig. 3b). While intra-somatic aSyn staining modestly increased throughout culture maturation, synaptic aSyn consistently increased with time in all neuronal subtypes, although more drastically in Hip neurons (Fig. 3c). Altogether, the ratio of synaptic to somatic aSyn increased with time, and was significantly higher in Hip compared to Cx neurons at DIV21 (Fig. 3d).

**Figure 4:**
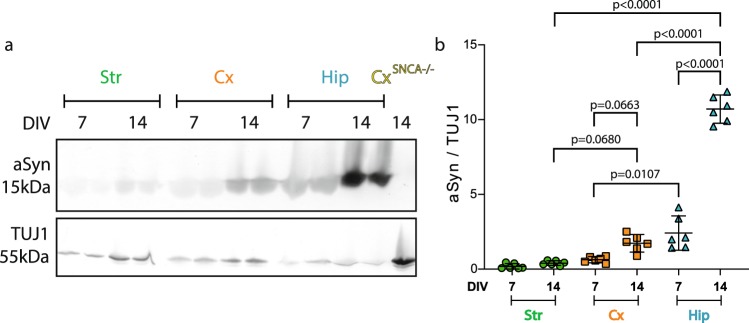
Relationship between aSyn expression level and pSyn accumulation in primary neurons. Primary neuronal cultures from WT or *SNCA*−/− embryos were lysed at DIV7 or 14 and the amount of aSyn and TUJ1 quantified by Western blotting. (**a**) A representative WB is shown. Full length gels are available in Supp. Fig. S10. (**b**) Quantification of aSyn signal normalized to TUJ1. 6 individual replicates from 3 individual experiments. Means +/− CI95 are shown. Two-way ANOVA was performed, followed by Tukey’s multiple comparisons test. Adjusted p values are shown.

We next quantified aSyn expression in DIV7 and DIV14 neuron cultures by Western blot. No aSyn could be detected in Cx^SNCA−/−^ neuron lysates (Fig. 4a). The amount of aSyn was normalized to TUJ1. aSyn expression increased in Cx and Hip cell cultures between day *in vitro* (DIV) 7 and DIV14, although only significantly in Hip cultures. The amount of aSyn in DIV7 Hip neurons was 3.8 and 12.7 fold higher than in Cx and Str neurons of similar age, respectively. After 14 days of culture, this difference in aSyn levels was even more important (6.2 and 26.4 fold, respectively) (Fig. 4b). Taken together, the side-by-side assessments of pSyn accumulation (Fig. 3) and aSyn expression (Fig. 4) within Hip, Cx and Str neurons strongly suggest that exogenous Fibrils seeding efficiency in neurons tightly depends on aSyn expression levels.

### aSyn expression level predicts pSyn deposition following exposure of neurons to exogenous aSyn Fibrils

**Figure 5:**
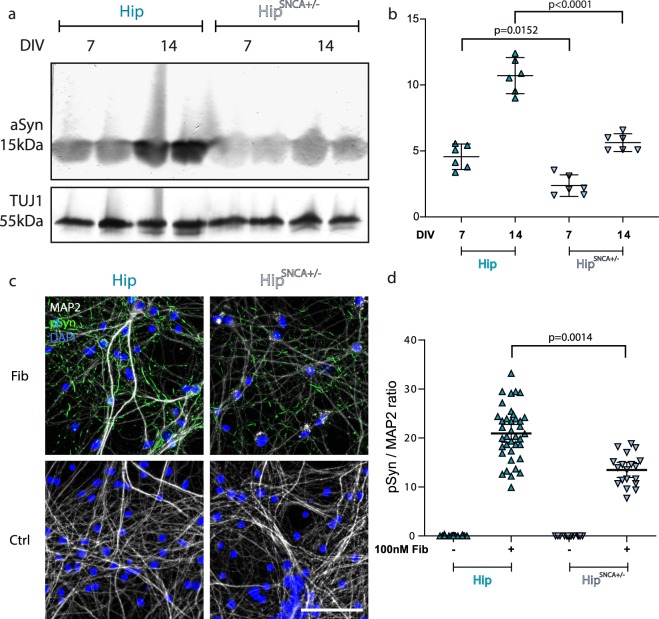
Decreased aSyn Fibrils seeding propensity upon reduction of endogenous aSyn expression levels. (**a**) Primary neuronal cultures from WT or *SNCA* +*/−* embryos were lysed at DIV7 or 14 and the amount of aSyn and TUJ1 quantified by Western blotting. A representative WB is shown. Full length gel is available in Supp. Fig. S11. (**b**) Quantification of aSyn signal normalized to TUJ1. Six individual replicates from 2 to 4 individual experiments. Means +/− CI95 are shown. Two-way ANOVA was performed, followed by Tukey’s multiple comparisons test. Adjusted p values are shown. (**c**) Cells were exposed to 100 nM aSyn Fibrils or vehicle at DIV7 and fixed at DIV21. Staining was performed for pSyn (green), MAP2 (grey) and Hoechst (blue). Scale bar represents 100 µm. (**d**) pSyn area/MAP2 area at fixed thresholds. 14 to 38 replicates from 3 to 4 individual experiments. Means +/− CI95 are shown. Two-way ANOVA was performed, followed by Tukey’s multiple comparisons test. Adjusted p values are shown.

**Figure 6:**
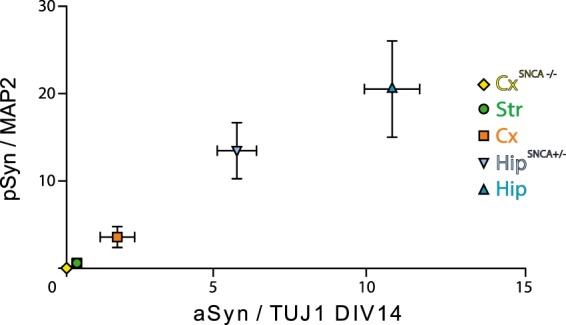
Dose-response relationship between exogenous aSyn Fibrils-mediated aSyn seeding and endogenous aSyn expression levels. The average aSyn expression level normalized to TUJ1 at DIV14 from Figs. 4 and 5 was plotted in x with standard deviation (SD) as error bars. The expression level of aSyn in Cx^*SNCA*−/−^ is 0. The average pSyn/MAP2 ratios in primary neuronal cultures from different brain regions exposed to exogenous aSyn Fibrils (data from Figs. 2 and 5) was plotted in y with SD as error bars.

Factors such as subcellular synuclein localization, neuronal proteostasis, and neuronal physiology, might also impact exogenous Fibrils seeding propensity. To strengthen our hypothesis of a link between aSyn expression levels and the extent of pSyn deposition following exposure of neurons to exogenous aSyn Fibrils, we selectively modulated aSyn expression levels by crossbreeding *SNCA*−/− and WT mice and generating Hip primary neurons from SNCA−/ + embryos. Assessment of aSyn expression levels normalized to TUJ1 expression in Hip *SNCA*+*/−* neurons was 52% that in WT Hip neurons at DIV7 and DIV14 (Fig. 5a,b). WT and *SNCA*+*/−* Hip neurons were next exposed to Fibrils and fixed as described previously. *SNCA*+*/−* Hip neurons exhibited less and smaller sized pSyn particles as compared to WT neurons (Fig. 5c). The pSyn/MAP2 ratio in Hip *SNCA*+*/−* exposed to Fibrils was 30% lower than that in WT Hip neurons (Fig. 5c,d). In addition, upon plotting the average pSyn/MAP2 ratio after exposure to Fibrils for 14 days, a clear correlation was observed with the expression of aSyn in the 5 different neuronal cultures we used (Fig. 6). Altogether, these results reinforce our conclusion that aSyn expression levels define the seeding propensity of exogenous Fibrils in different neuronal cell populations.

## Discussion

The spread and amplification of protein aggregates is believed to contribute to the progression of several neurodegenerative diseases^5,56–60^, in particular PD. The binding of aSyn assemblies to neuronal cell membranes is key to their prion-like propagation process. To this end, we previously identified aSyn assemblies neurons and astrocytes membranous interactome^31^. While the membranous interactome of aSyn assemblies, as demonstrated by their differential binding to different brain cells^61^, may define the tropism of aSyn assemblies for distinct neuronal cells, what defines their amplification remains elusive. Here, we show that despite a comparable uptake of exogenous aSyn Fibrils (Fig. 1), the extent to which endogenous aSyn is seeded depends on the origin of the primary neurons (Fig. 2). Most importantly, we establish a relationship between aSyn expression levels and the seeding efficiency of exogenous aSyn Fibrils. This is achieved through a careful and quantitative assessment of aSyn expression level and the amplification of exogenous seeds in 5 different primary neuronal cultures from Striatum, Cortex and Hippocampus of WT (*SNCA*+/+), aSyn knock out (*SNC*A −/−) and heterozygous (*SNCA*+*/−*) mice (Figs. 2–6). Altogether, our results demonstrate that aSyn expression levels define the vulnerability of neuronal cell populations to seeding.

Our findings are in good agreement with the literature on PD showing that changes in aSyn expression levels affects LP progression in patients. Indeed, duplications or triplications of the *SNCA* gene encoding aSyn were shown to be responsible for the early onset of familial forms of PD, with *SNCA* triplication inducing a more severe form of the disease than duplication^62–64^. Data obtained from pathological human brains suggested a relative good correlation of regional LP burden with aSyn expression, with some exceptions^50^. The imperfect agreement between LP burden and aSyn expression reported in human brain regions may be the consequence of the cellular heterogeneity, the complex topography of polarized neurons, or preferential antero- or retrograde active transport of aggregated aSyn and its local accumulation in the *in vivo* 3D environment. Indeed, as aSyn is mostly presynaptic, its local concentration could parallel that of afferents. In addition, aSyn aggregates appear in a centripetal fashion in neurons^65^, in agreement with our findings where pSyn deposits accumulate in the somata of neurons exposed to exogenous Fibrils, despite aSyn expression in the presynaptic terminals of mature neurons (Fig. 4) (Supp. Fig. S4).

Importantly, aSyn expression levels reported in this study seem to match the ones available in online resources. Indeed, murine SNCA mRNAs abundance reported on the Allen Mouse Brain Atlas website^66^ (accessible at http://mouse.brain-map.org/gene/show/20379) correlates well with the protein expression level we quantified in this study by WB and image analysis (Supp. Fig. S7, Figs. 3 and 4). While SNCA mRNAs are almost absent from Str neurons, they are found in Cx neurons and are very abundant in Hip neurons in adult mice. The normalized expression quantification available on the same page yet indicates a comparable expression level in Hip and Cx, which we attribute to a normalization of expression by the whole regions areas, which includes neuronal processes from other regions. This normalization would thus not reflect the very high SNCA abundance in Hip neurons. Data available for human brains on the Allen Human Brain Atlas^67^ (accessible at http://human.brain-map.org/microarray/gene/show/6582) suggest that in humans, SNCA mRNA abundance is slightly higher in Cx than in Hip. It is however much lower in the caudate-putamen region. As human data do not allow the visualisation of the spatial distribution of mRNAs abundance,  which is available for mouse data, we suppose that they are once again normalized to the region area, and thus might not reflect the very high concentration of SNCA mRNA in single neurons. Moreover, it is important to note that these data focus on mRNA abundance, and that aSyn protein translation and degradation rates might significantly differ between these regions. Thus, as SNCA mRNA abundance might differ in human and murine brain regions, the patterns of aSyn expression levels might also differ, with as a consequence a differential regional aggregation propensity upon focal exposure to seeding aggregates.

It is worth noting that a relationship between aSyn expression levels and pSyn accumulation in different neuronal subtypes in the hippocampus has been proposed^52,53^. aSyn expression levels definitely impact the seeding efficiency of exogenous Fibrils as they define not only the rate at which exogenous endocytosed seeds grow by incorporating endogenous aSyn, but also the persistence of those aggregates despite the cellular machinery in charge of clearing them. Indeed, neurons expressing the highest levels of aSyn fuel better the ongoing seeded aggregation, thus allowing a rapid growth that outweighs their clearance.

LP development in different regions of the mouse brain, after *in vivo* injection of fibrillar assemblies, shows clear similarities with the differential accumulation of pSyn we report using primary neuronal cultures from Str, Cx and Hip. Indeed, Luk and colleagues^14^ reported that injection of fibrillar assemblies in the striatum of WT mice resulted in moderate pSyn accumulation in the injected region. The injection of the same fibrillar assemblies in the cortical or hippocampal regions resulted in severe pSyn pathology. Intriguingly, although their pSyn accumulation capacity upon exposure to exogenous Fibrils was the highest among the brain regions we analysed, hippocampal neurons are not among the earliest to be affected by LP in PD patients. Indeed, the hippocampus is affected by LP pathology only at Braak stage 3^10^. We propose that the discrepancy between *in vivo* and *in vitro* data is the consequence of the progressive prion-like spread to Hip neurons of aSyn aggregates from other brain regions affected earlier. These regions might have special characteristics allowing *de novo* aggregation of aSyn, such as an intrinsic high oxidative stress, exposure to environmental stress, a transient increase in aSyn expression, etc. as reviewed by James Surmeier in 2017^48^. Numerous experiments from the literature strongly support that neuroanatomical connectivity is another central parameter generating a specific spread pattern. Indeed, focal injection of aSyn assemblies in the striatum of mice is followed by direct retrograde propagation to neurons in distant regions, in a manner which can be partially modelled by both the abundance of their axonal projections to the injection point, and by endogenous aSyn expression levels^68^. At later time points, aSyn aggregation has been suggested to spread in an anterograde manner to neurons which do not project to the injection point^69^. In principle, the experimental paradigm we designed appears perfectly suited to assess the dependence of the exogenous Fibrils seeding propensity on aSyn expression within neurons from regions that are affected early on by pSyn deposits, such as dopaminergic neurons from the substantia nigra, provided they are amenable to be grown in sufficient amounts *in vitro*. Unfortunately, this is not yet the case, as rodent primary mesencephalon cultures typically contain only a low percentage of dopaminergic neurons^70^.

It is worth highlighting that the dependence of the exogenous Fibrils seeding propensity on aSyn expression within specific neuronal populations cannot be relevantly addressed in transgenic models where aSyn expression is driven through the use of a non-SNCA promoter, such as the Prion promoter^51^. Similarly, extreme care should be taken when analysing the results of (exogenous) human aSyn (haSyn) expression in mice on a WT background. Indeed, as co-expressing murine aSyn (maSyn) with haSyn decreases haSyn aggregation^36,71^, the seeding propensity of haSyn fibrils will also be highly dependent on the level of maSyn in different mouse brain regions.

## Conclusions

By using primary neuronal cultures from various brain regions of mice expressing different levels of aSyn, we demonstrated that neuronal populations exhibited strong differences in their ability to template aSyn aggregation after exposure to exogenous aSyn Fibrils. We demonstrate that exogenous aSyn Fibrils seeding propensity depends on neuronal aSyn expression levels. Our observations bring new insights into the mechanisms underlying specific vulnerability of brain regions and neuronal subtypes to LP, thus providing a comprehensive framework for the spatiotemporal spread pattern of pSyn within the central nervous system.

## Methods

### Microfluidic culture devices preparation

Primary neuronal cultures were established in custom-made microfluidic chips, for a diminished consumption of cells and reagents per replicate and longer culture duration. These microfluidic culture chips were made of a polydimethylsiloxane (PDMS, Dow Corning) part, bonded after plasma treatment to a microscope compatible glass coverslip (Dutscher). PDMS cell culture platforms were 800 µm wide, 4 mm long and 50 µm high rectangular chambers, and accessible through two inlets. Master mould preparation, plasma bonding and surface coating of microfluidic chips by 10 µg/mL poly-D-lysine were performed as previously described^49^. Culture systems were then additionally coated with 2.5 µg/mL of laminin (Sigma) for 6 hours.

For biochemical analysis, neurons were plated in conventional 24-wells culture systems (VWR), coated following the same protocol as microfluidic culture devices.

Microfluidic chips for co-culturing neuronal populations in two separate chambers connected with microchannels for directed axonal growth (see sketch in Supp. Fig. S6a) were prepared as previously described^49^.

### Primary neuronal cultures

Animal care was conducted in accordance with standard ethical guidelines (U.S. National Institutes of Health publication no. 85-24, revised 1985, and European Committee Guidelines on the Care and Use of Laboratory Animals) and the local, IBPS and UPMC, ethics committee approved the experiments (in agreement with the standard ethical guidelines of the CNRS “Formation à l′Expérimentation Animale” and were approved by the “C2EA −05 Comité d’éthique en experimentation animale Charles Darwin”). Swiss and C57BL6N mice E14.5-E16.5 embryos (Janvier Labs) were used for WT primary cultures generation. For *SNCA*−/− cell culture generation, we used embryos from C57BL6jOlahsd mice (Envigo) exhibiting a spontaneous deletion of the SNCA locus^72^. *SNCA*+*/−* embryos were obtained by crossbreeding C57BL6N and C57BL6jOlashd mice.

Structures obtained by microdissection were rinsed with Gey’s Balanced Saline Solution (Thermo Fisher). They were then incubated in Dulbecco’s Modified Eagle Medium (DMEM) (Life Technologies) containing papain (Sigma) for 10 minutes at 37 °C. Enzymatic activity was then inhibited by addition of 10% foetal calf serum (FCS, GE Healthcare). Enzyme-containing solution was replaced by DMEM containing DNase type I (Sigma) and structures were mechanically dissociated with a pipette. After several rounds of rinsing, cells were re-suspended in culture medium (91% High glucose Glutamax supplemented DMEM, 5% FCS, 1% Penicillin/Streptomycin 1000U/ml, 1% N2 Supplement and 2% B27 Supplement (Life Technologies)) at 20 million cells per ml. 1.5 µl of cell suspension was injected in microfluidic cell culture chambers, and left to adhere for a few minutes before cell culture medium addition. Microfluidic chips were stored in a petri dish containing 0.5% Ethylene-diamine-tetra-acetic acid in water, allowing for reduced evaporation of culture medium while preventing bacterial growth.

Cells were maintained at 37 °C, 5% CO2 in a humidified atmosphere, and half of the medium was renewed every week. Culture medium was completely renewed when treatment was performed.

### Fibrils preparation

The expression, purification of mouse monomeric WT aSyn and its assembly into the fibrillar polymorph Fibrils were carried out as previously described^35,73^. aSyn Fibrils in 50 mM Tris-HCl, pH 7.5, 150 mM KCl were pelleted and washed with PBS buffer, pH 7.4 twice. aSyn Fibrils were labelled with a 2 molar excess of NHS-ester ATTO 550 (AttoTec) according to the manufacturer’s recommendations. The labelling reaction was stopped by addition of Tris, pH 7.5 to a final concentration of 1 mM. The unreacted fluorophore was removed by two cycles of centrifugations at 15,000 g for 10 minutes and resuspension of the pelleted fibrils in PBS, pH 7.4. The fluorescently labelled aSyn Fibrils were fragmented by sonication for 20 min in 2-ml Eppendorf tubes in a Vial Tweeter powered by an ultrasonic processor UIS250v (250 W, 2.4 kHz; Hielscher Ultrasonic, Teltow, Germany). The fragmented, fluorescently labelled, aSyn Fibrils were flash frozen in liquid nitrogen and stored at −80 °C until they were used. All aSyn fibrillar assemblies were imaged before and after fragmentation by transmission electron microscopy after adsorption of 200 mesh carbon coated electron microscopy grids and negative staining with 1% uranyl acetate. Length distribution of the fragmented fibrils were performed and are represented as histograms where the number of fibrils analysed is indicated.

### Cells exposure to fibrils

The frozen, fragmented, fluorescently labelled, aSyn Fibrils were thawed in a 37 °C water bath immediately before use and extemporaneously diluted in neuronal cell culture medium. All procedures were conducted under a vertical laminar flow culture hood and Fibrils containing liquids as well as potentially contaminated surfaces and objects were treated/cleaned with a 2% sodium dodecyl sulphate solution, as recommended in^74^. Control condition corresponds to the exposure of cells to fresh medium, as the volume of vehicle (PBS) is negligible (0.1%) in cultures treated with 100 nM of Fibrils. It has previously been shown that monomeric aSyn species have no seeding propensity even at the highest concentration used in this work (500 nM)^75^.

### Cells fixation and staining

Cells were fixed with a 4% paraformaldehyde (PFA, Electron Microscopy Science), 4% sucrose (Sigma) phosphate buffered saline (PBS, Thermo Fisher) solution. Saturation and permeabilization were performed simultaneously with a 1% bovine serum albumin (BSA, Sigma) 0.2% Triton X-100 (Sigma) PBS solution for 30 min at RT. Antibodies were diluted in 1% BSA PBS solution. Primary antibodies were incubated overnight at 4 °C. C20R antibody from Abcam diluted at 1/1000 was used to stain endogenous aSyn, antibodies 59264 and 51253 from Abcam were used to stain phosphorylated aSyn (pSyn), anti-microtubule associated protein 2 (MAP2) HM-2 clone antibody from Sigma was used at 1/500, anti-ß-III-tubulin (TUJ1) clone SDL.3D10 from Sigma was used at 1/1000, anti-CD68 clone FA-11 from Abcam was used at 1/1000 to stain microglia, and anti-GFAP Z0334 from Dako was used at 1/500 to stain astrocytes. Secondary antibodies coupled to Alexa Fluor 488, 555 or 633 (Thermo Fisher) were used at 1/500 and incubated 1 hour and a half at room temperature. Hoechst probe (Sigma) was incubated at the same time as secondary antibodies, at a concentration of 1/5000. Cells were washed between each step with a 1% BSA PBS solution, and were stored in a 0.1% azide (Sigma) PBS solution for preventing bacterial growth.

### Image acquisition and analysis

Image acquisition was performed with an Axio-observer Z1 (Zeiss) fitted with a 20X N.A. 0.5 objective (Zeiss) and a cooled CCD camera (ORCA-Flash 4 (Hamamatsu), Coolsnap HQ2 (Roper Scientific), Metamorph software (Molecular Imaging)). On average, 5 random visual fields were acquired per microfluidic culture chamber. Image analysis was performed with Fiji software^76^. In order to have all objects in focus in each field, 4 acquisitions were made, each distant by 2um in z. Images were then combined using the Z project function of Fiji. In order to normalize background signal, images were pre-treated with the Subtract Background function of Fiji, radius 500 pixels for MAP2 signal and 5 pixels for pSyn signal.

pSyn neuronal burden analysis was performed by computing the ratio of pSyn area over MAP2 area at fixed thresholds.

aSyn subcellular localization analysis was performed by measuring the integrated density of the aSyn signal inside or outside of somatic compartments, manually delimited in the same field from MAP2 staining.

GFAP area occupancy was quantified by thresholding the GFAP signal at a fixed value for all pictures and computing the percentage of the field occupied by the binarized signal.

Percentage of cells expressing specific markers, as well as percentage of condensed nuclei, were manually evaluated.

Confocal imaging was performed on a Leica SP5 fitted with a 63X N.A. 1.4 objective.

### Fibrils uptake analysis

Because fixation with PFA strongly diminished Fibrils fluorescence (Supp. Fig. S8), cells were imaged live with an epifluorescence microscope. Cells were treated with 20 or 100 nM of Fibrils at DIV7, washed twice with culture medium 24 hours after treatment and immediately imaged. 5 fields were taken for each replicates. Fibrils associated fluorescence signal was measured in regions of interest (somata and a few µm of proximal neurites.), which were drawn from phase contrast acquisitions.

### Western blot

Cells grown in 24-well plates were directly lysed and homogenized under reducing conditions in 4x Laemmli buffer (about 25 µL/well) on ice using a cell scraper, transferred to an Eppendorf tubes, heated to 95 °C for 4 min and vigorously vortexed. After cooling to room temperature, samples were supplemented with solid urea up to a final concentration of ~5 M, then frozen at −20 °C until further use. Electrophoresis was performed on ultrathin (100 µm) 12% polyacrylamide gels^77^. After transfer onto nitrocellulose membrane (Protran 0.45 µm, GE Healthcare), the lower molecular weight part of the Western blot (i.e. the region corresponding to aSyn bands) was additionally fixed in 4% PFA/0,05% glutaraldehyde in PBS for 30 min (adapted from^78^). After extensive washing in PBS, the membrane was blocked with 5% fat-free milk powder in PBS (for aSyn detection), or 5% milk powder in TBS supplemented with 50 mM NaF for pSyn detection, and incubated overnight with the different primary antibodies in the same solution. The C20R antibody (Santa Cruz) was used for detecting aSyn at 1/1000; for pSyn detection we used the D1R1R antibody from Cell Signaling Technology at 1/300, instead of the pSyn antibodies used for immunocytochemistry. The upper part of the Western blot was reacted with MMS-435P antibody against TUJ1 at 1/15000 (Biolegend). Alkaline phosphatase-coupled secondary antibodies were from Jackson ImmunoResearch (1/5000), and detection performed using nitro blue tetrazolium chloride/5-bromo-4-chloro-3-indolyl-phosphate reagent (NBT/BCIP) in Tris-Mg^++^ buffer at pH 9.5. After drying, Western blots were scanned, and relative protein amounts evaluated using the ImageStudio^TM^ Lite application (Li-Cor Biosciences), with TUJ1 band intensities as internal reference for total neuronal protein content. When possible, the transfer membrane was cut in half to reveal loading control and the protein of interest of known molecular weights separately.

### Statistical analysis

Statistical tests were performed with GraphPad Prism 8. For experiments where two factors were modified (such as cell type plus culture age), overall statistical significance was assessed with a two way ANOVA test, and Tukey’s multiple comparisons were then performed. For one experiment where only one factor was modified (Fig. 2c, cell type), normality was assessed with a Shapiro-Wilk test, followed by a one way ANOVA test and Dunnett’s T3 multiple comparisons test.

In this work, an individual experiment corresponds to the generation of one batch of primary cultures from embryos extracted from a single pregnant mouse. Each individual replicate then corresponds to an individual culture chamber, seeded with cells and treated separately from the other ones. When individual cells were analysed, such as in Fig. 2c, a minimum of 50 neurons were considered for each individual culture chamber.

While individual replicates are shown on the graphs, statistical tests were performed on mean results from a minimum of 3 individual experiments.

## Supplementary information

Supplementary figures and legends.

## Data Availability

The datasets generated during the current study are available from the corresponding author on reasonable request.
