# Supplementary material for: The expression level of alpha-synuclein in different neuronal populations is the primary determinant of its prion-like seeding
Source: Sci Rep. 2020 Mar 17;10:4895. doi: 10.1038/s41598-020-61757-x (PMC7078319; doi:10.1038/s41598-020-61757-x)
Supplement: Supplementary file 1 — Supplementary figures and legends. [file 41598_2020_61757_MOESM1_ESM.pdf]

The expression level of alpha-synuclein in different neuronal populations is the primary determinant of its prion-like seeding.

Supplementary Information

Courte J[1,3], Bousset L[2‡], Von Boxberg Y[1‡], Villard C[3], Melki R[2], Peyrin JM[1*]

[1] Sorbonne Universités, Faculté des Sciences et Technologie, CNRS UMR 8246, INSERM U1130, Neurosciences Paris Seine, Institut de Biologie Paris Seine, Paris, 75005, France.

[2] Laboratory of Neurodegenerative Diseases, Institut François Jacob, MIRCen, CEA-CNRS, Fontenay aux Roses 92265, France

[3] Physico-Chimie Curie, Université PSL, CNRS, Institut Pierre-Gilles de Gennes pour la Microfluidique, Paris, France.

‡ Equal contribution

* Corresponding author

Supplementary Figure S1

Characterization of primary neuronal cultures with cellular identity markers.

Primary neuronal cultures were fixed at DIV14, before being stained and imaged with an epifluorescence microscope. **(a)** Representative fields of Str, Cx and Hip neurons stained for DAPI (red) and NeuN (green). Scale bar represents 100µm. **(b)** Quantification of the percentage of nuclei co-localized with NeuN signal. 3 to 6 replicates from 1 to 2 individual experiments. Means +/- standard deviation (SD) are shown. **(c)** Representative fields of Str, Cx and Hip neurons stained for DAPI (red) and GFAP (green). Scale bar represents 100µm. **(d)** Quantification of the area percentage occupied by the GFAP signal binarized with a fixed threshold. 3 to 6 replicates from 1 to 2 individual experiments. Mean +/- CI95 are shown. **(e)** Representative fields of Str, Cx and Hip neurons stained for NeuN (red) and GAD67 (green). Scale bar represents 100µm. **(f)** Quantification of the percentage of NeuN+ cells co-localized with GAD67 signal. 2 to 6 replicates from 1 to 3 individual experiments. Mean +/- CI95 are shown.

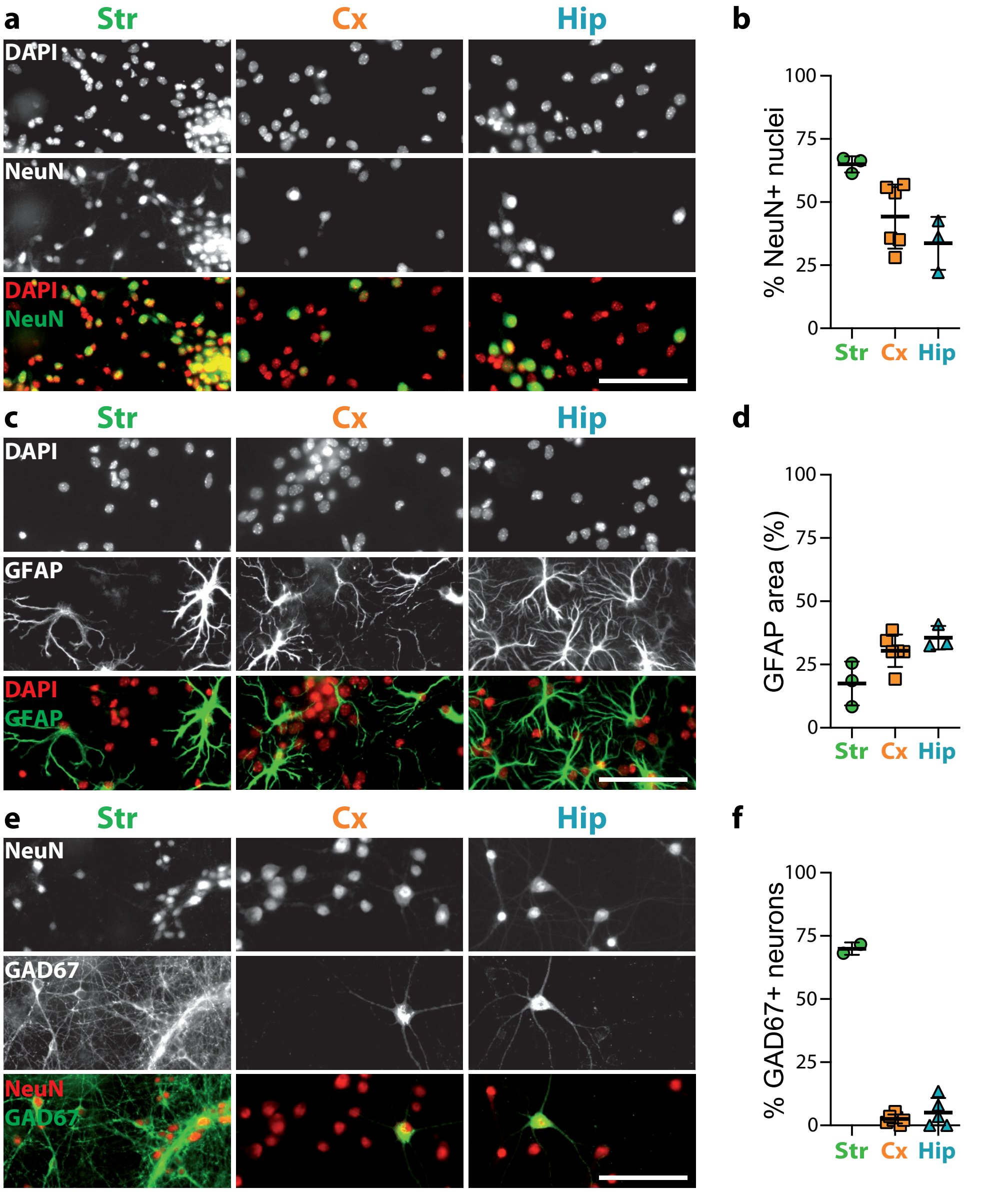
a
Str
Cx
Hip
DAPI
NeuN
DAPI
NeuN
b
% NeuN+ nuclei
100
75
50
25
0
Str
Cx
Hip
c
Str
Cx
Hip
DAPI
GFAP
DAPI
GFAP
d
GFAP area (%)
100
75
50
25
0
Str
Cx
Hip
e
Str
Cx
Hip
NeuN
GAD67
NeuN
GAD67
f
% GAD67+ neurons
100
75
50
25
0
Str
Cx
Hip

Supplementary Figure S2

Relationship between exogenous aSyn fibrils amounts and endogenous aSyn seeding.

Primary hippocampal cultures were exposed to increasing concentrations of exogenous aSyn Fibrils at DIV7 and imaged 14 days later. **(a)** Representative fields. Cells were stained for MAP2 (grey) and pSyn (green). Scale bar represents 55µm. **(b)** pSyn area / MAP2 area ratio. 3 to 6 individual replicates. Mean +/- CI95 are shown. **(c)** Percentage of neurons harbouring somatic pSyn assemblies. 3 to 6 individual replicates. Mean +/- CI95 are shown.

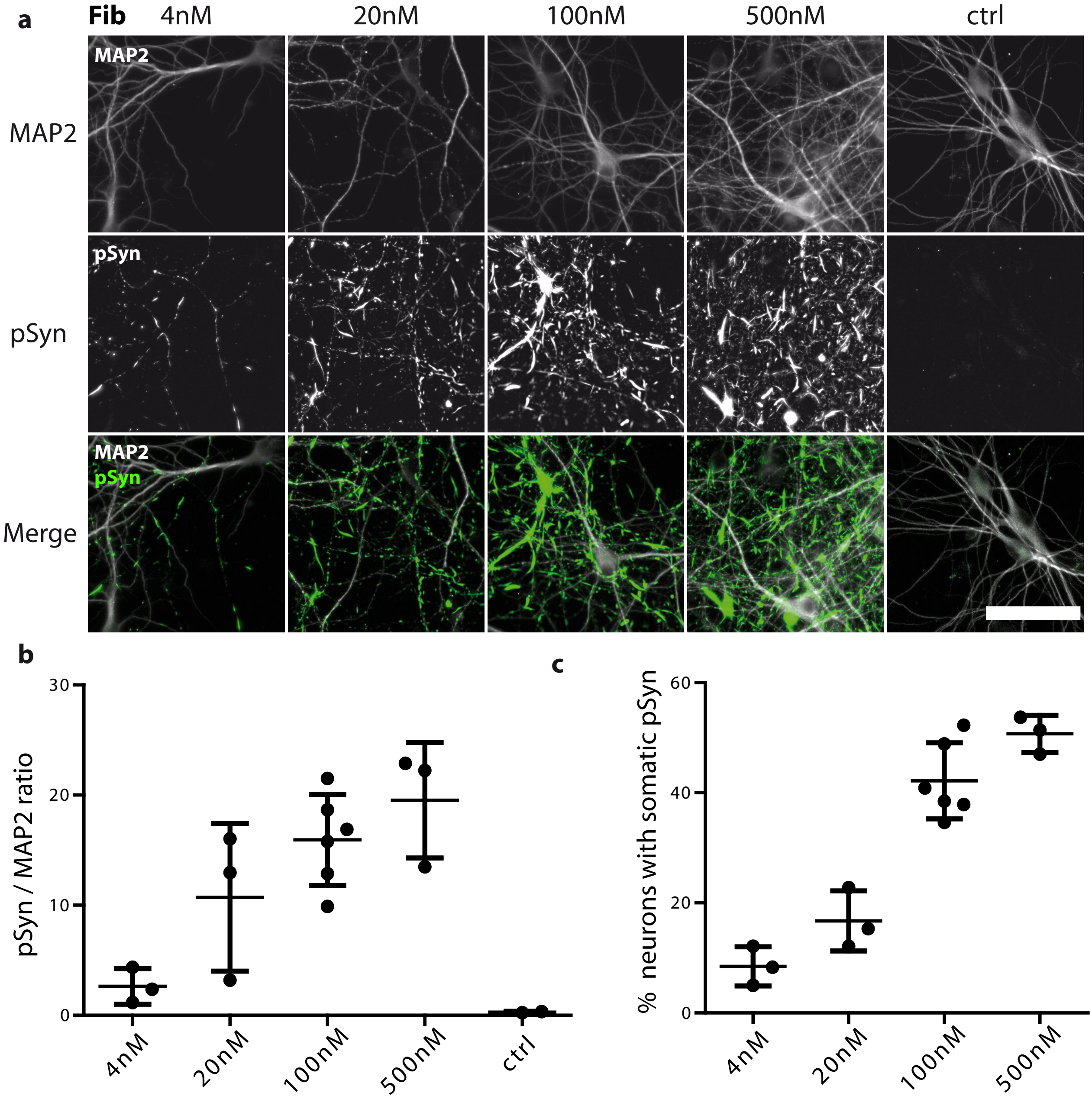
a
Fib
4nM
20nM
100nM
500nM
ctrl
MAP2
pSyn
Merge
MAP2
pSyn
b
pSyn / MAP2 ratio
30
20
10
0
4nM
20nM
100nM
500nM
ctrl
c
% neurons with somatic pSyn
60
40
20
0
4nM
20nM
100nM
500nM

Supplementary Figure S3

Characterization of the exogenous aSyn Fibrils used throughout this work.

Transmission electron micrographs of negatively-stained murine aSyn Fibrils **(a)** before and **(b)** after fragmentation. Scale bar represents 200nm. **(c)** Length distribution of the fragmented aSyn Fibrils. The number (n) of fibrils the histograms were derived from is indicated.

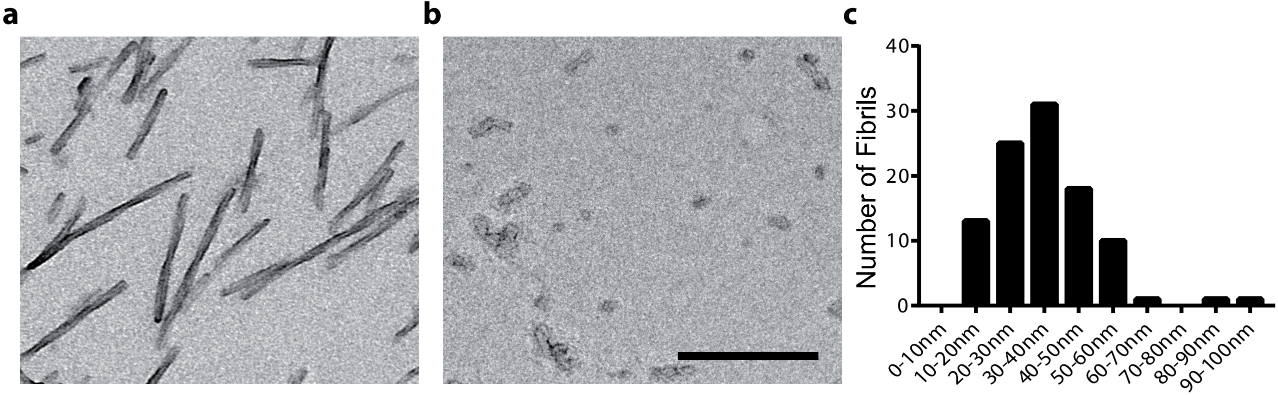
a
b
c
Number of Fibrils
40
30
20
10
0
0-10nm
10-20nm
20-30nm
30-40nm
40-50nm
50-60nm
60-70nm
70-80nm
80-90nm
90-100nm

Supplementary Figure S4

Co-localization of aSyn Fibrils and pSyn signals.

Primary hippocampal cultures were exposed to 100nM aSyn Fib at DIV7 and imaged 14 days later. MAP2 (grey) and pSyn (green) staining were performed. While aSyn Fibrils signal (red) was punctiform and mostly present in the soma of neurons and in non-neuronal cells, pSyn deposits were localized both in neuritic process and somas in the most extreme cases, forming perinuclear “cages”. Note the limited co-localization of pSyn and the most intense exogenous aSyn Fibrils signals. Scale bar represents 50µm.

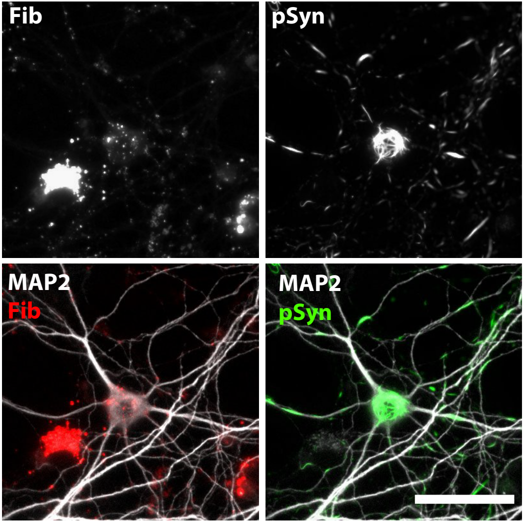
Fib
pSyn
MAP2
Fib
MAP2
pSyn

Supplementary Figure S5

Lack of significant cellular death in cultures exposed to exogenous aggregates.

Primary neuronal cultures were exposed to 100nM aSyn Fib at DIV7 and imaged 14 days later. Hoechst staining was performed. **(a)** Representative fields. Scale bar represents 100µm. **(b)** Quantification of the percentage of condensed nuclei. 8 to 13 replicates from 1 to 3 individual experiments. Mean +/- CI95 are shown. Two-way ANOVA was performed, followed by Tukey's multiple comparisons test. Adjusted p values are shown.

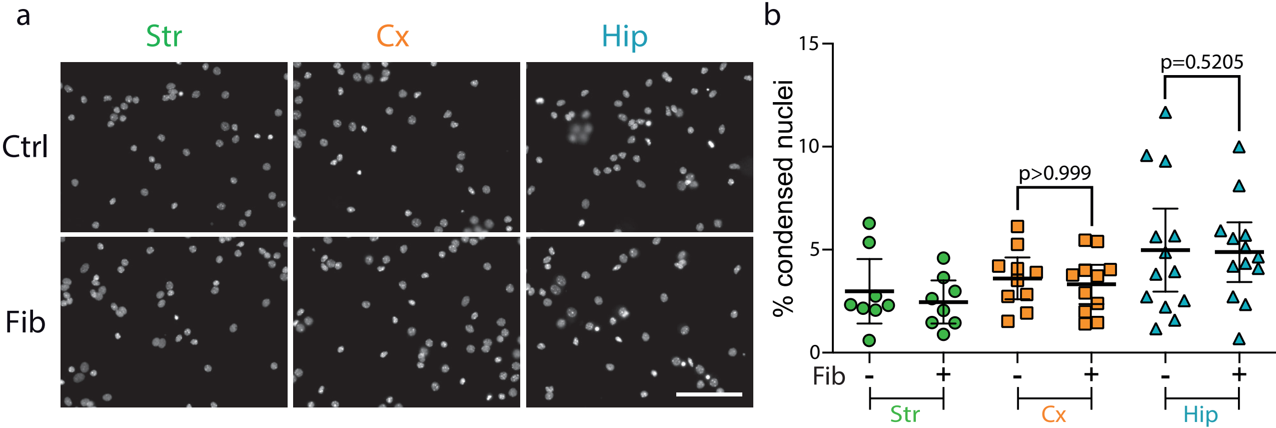
a
Str
Cx
Hip
Ctrl
Fib
b
% condensed nuclei
15
10
5
0
p>0.999
p=0.5205
Fib
-
+
-
+
-
+
Str
Cx
Hip

Supplementary Figure S6

Co-culture of Str with Cx$^{SNCA-/-}$- axons does not strongly impact pSyn accumulation after exposure to aSyn Fibrils.

Co-culture of Str neurons with Cx$^{SNCA-/-}$ axons was performed in a microfluidic co-culture chamber allowing for the preferential growth of axons from one side to another (here from the Cx$^{SNCA-/-}$ chamber to the Str chamber). Exogenous aSyn Fibrils were introduced at DIV7 in the Str chamber, and were selectively excluded from the Cx$^{SNCA-/-}$ chamber by maintaining a 100µl volume excess in the Cx$^{SNCA-/-}$ chamber. **(a)** Schematics of the experimental setup. In grey are the Cx$^{SNCA-/-}$ neurons and in red the Str neurons. **(b)** Representative fields of unconnected and connected Str neurons. Staining was performed for TUJ1 (grey), MAP2 (red), pSyn (green) and nuclei were stained with DAPI (blue). Scale bar represents 50µm. **(c)** pSyn area / MAP2 area ratio was computed. 4 to 5 individual replicates. Mean +/- CI95 are shown.

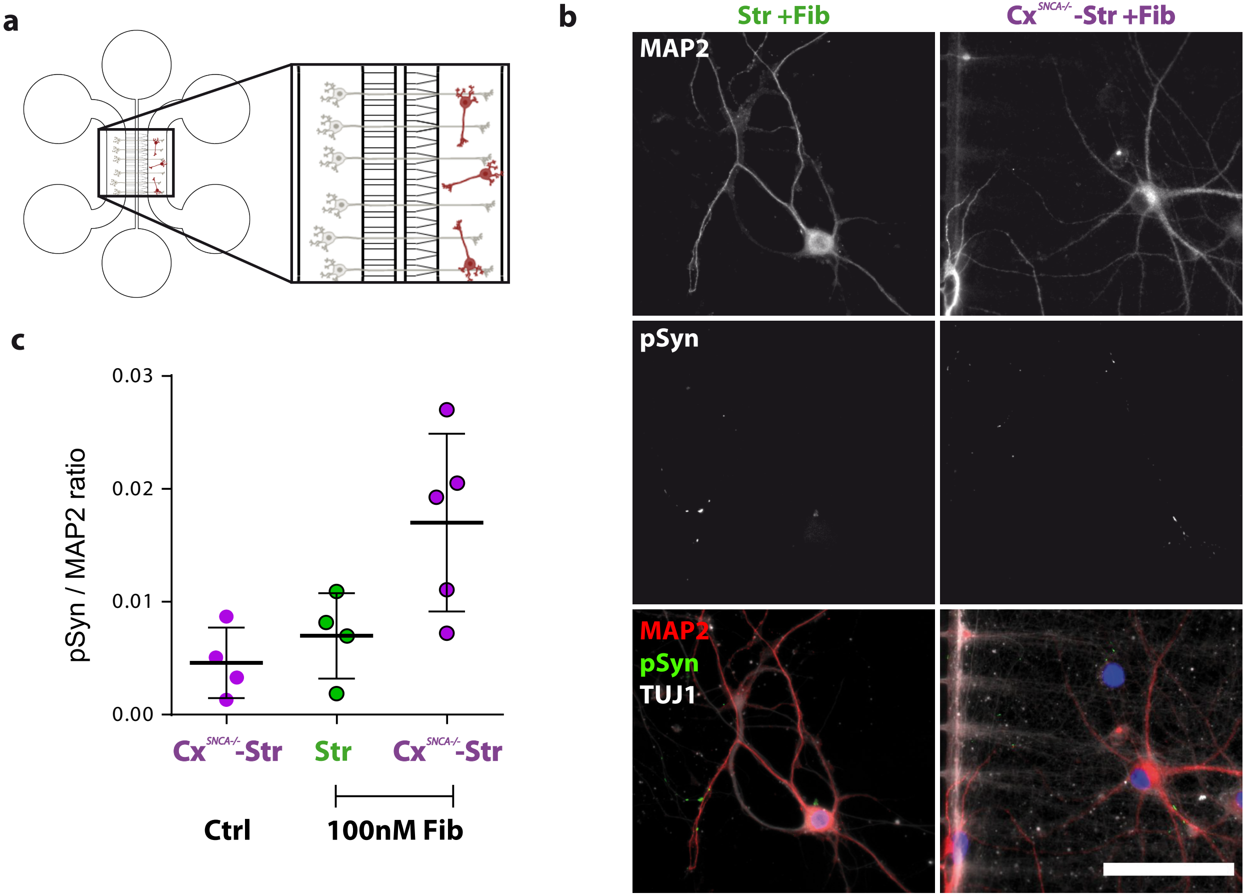
a
b
Str +Fib
Cx^SNCA-/- -Str +Fib
MAP2
pSyn
MAP2
pSyn
TUJ1
c
pSyn / MAP2 ratio
0.03
0.02
0.01
0.00
Cx^SNCA-/- -Str
Str
Cx^SNCA-/- -Str
Ctrl
100nM Fib

Supplementary Figure S7

SNCA mRNAs abundance in adult mouse brain regions overlaps with aSyn expression level in dissociated cultures.

Data on SNCA mRNAs dosage by *in situ* hybridization were obtained from the Allen Mouse Brain atlas website, at the following address: http://mouse.brain-map.org/gene/show/20379. Image 17 from the "Snca - RP_071218_03_E03 - coronal" dataset with the "Expression" filter (from low to high expression: blue-green-yellow-red), is depicted here. Brain regions, obtained from image 71 of the Allen Reference Atlas for coronal slices at the following address: http://mouse.brain-map.org/experiment/thumbnails/100048576 were drawn to visualize their relative abundance in SNCA mRNAs.

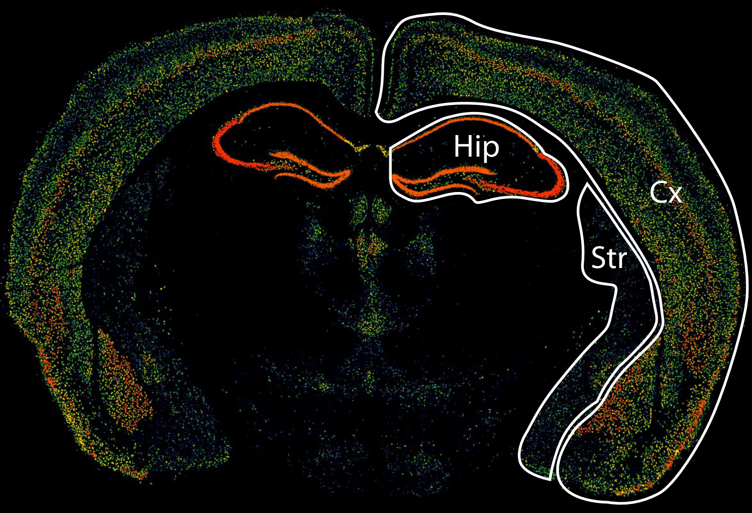
Hip
Cx
Str

Supplementary Figure S8

The fixation and permeabilization protocols decrease exogenous aSyn Fibrils fluorescence.

Primary hippocampal cultures were exposed to 100nM aSyn Fibrils at DIV7 and imaged 7 days later. Use of PBS versus culture medium did not notably modify signal to noise ratio of exogenous aSyn Fibrils fluorescence. 15min fixation with PFA notably decreased aSyn Fibrils fluorescence. 30min permeabilization with 0.2% Triton X-100 decreased signal even more and suppressed diffuse fluorescent signal that had appeared after PFA treatment. Scale bar represents 100µm.

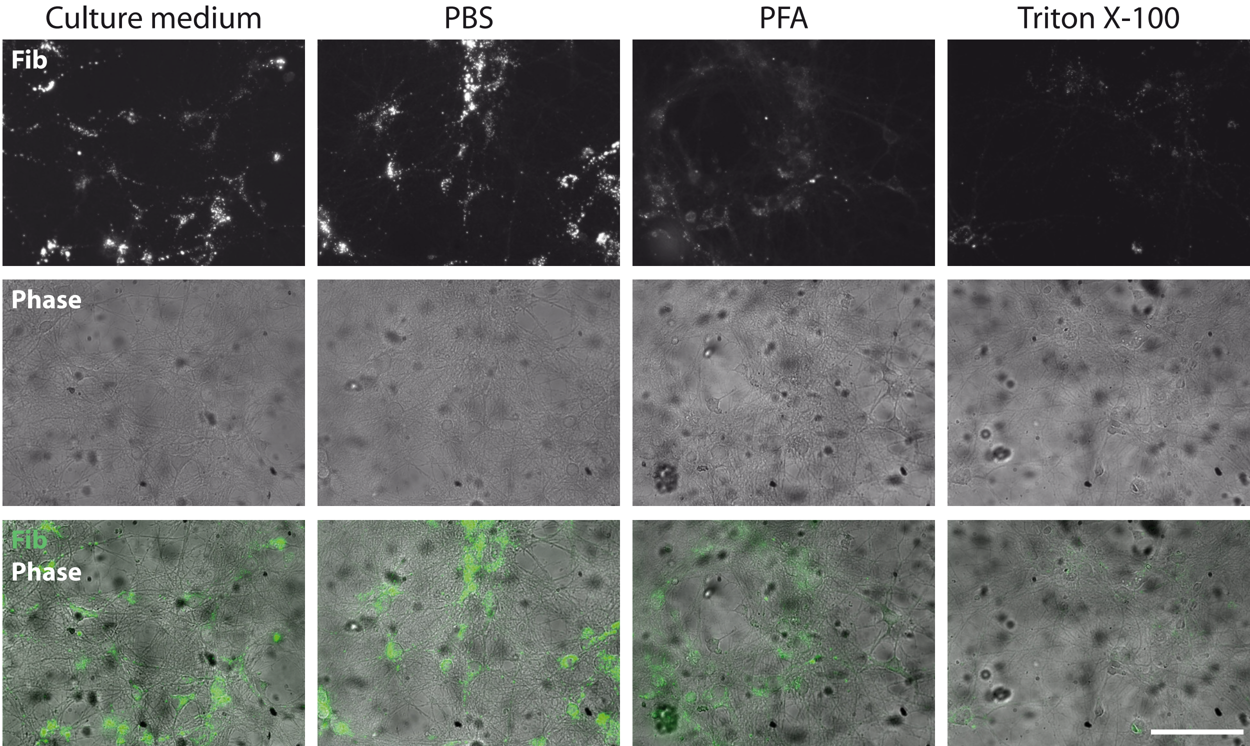
Culture medium
PBS
PFA
Triton X-100
Fib
Phase
Fib
Phase

Supplementary Figure S9

Full-length gels for Figure 2d.

Primary neuronal cultures exposed or not to 100nM of aSyn Fibrils at DIV7 were lysed at DIV14, and lysates were submitted to gel electrophoresis. Transfer membranes were revealed for **(a)** pSyn and **(b)** TUJ1. Molecular weights markers are partially cropped but visible on the rightmost side of (a). Lanes 15 to 18 (rightmost) of (a) corresponding to lanes 13 to 16 of the loading control (b) were not relevant to this publication, and thus not annotated. They were cropped out of Figure 2d.

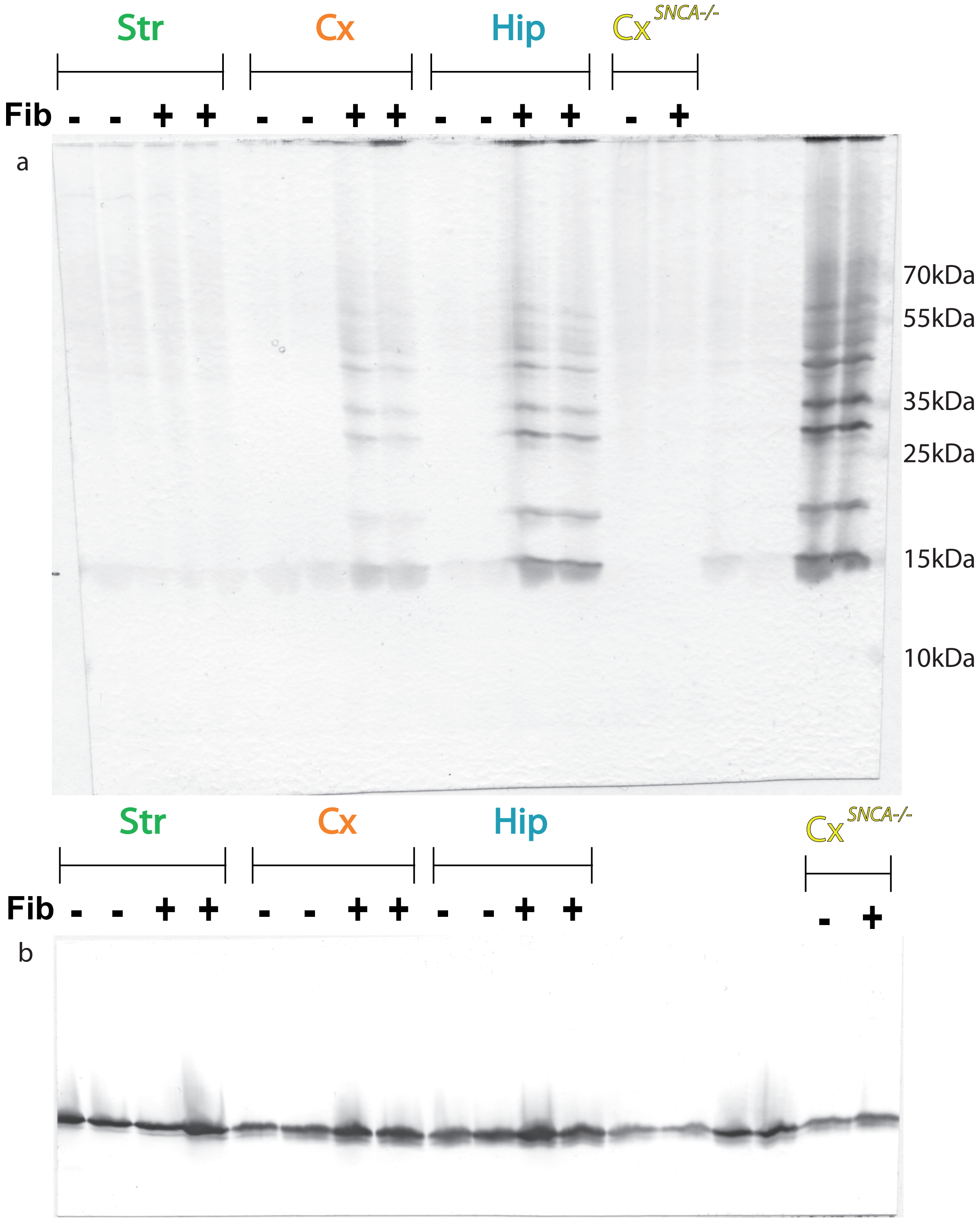

Str
Cx
Hip
Cx SNCA-/-
Fib - - + + - - + + - - + + - +
a
70kDa
55kDa
35kDa
25kDa
15kDa
10kDa
Str
Cx
Hip
Cx SNCA-/-
Fib - - + + - - + + - - + +
- +
b

Supplementary Figure S10

Full-length gels for Figure 4b.

Primary neuronal cultures exposed or not to 100nM of aSyn Fibrils at DIV7 were lysed at DIV7 or DIV14, and lysates were submitted to gel electrophoresis. Transfer membranes were revealed for **(a)** aSyn and **(b)** TUJ1. Molecular weights markers are partially cropped but visible on the rightmost side of (a).

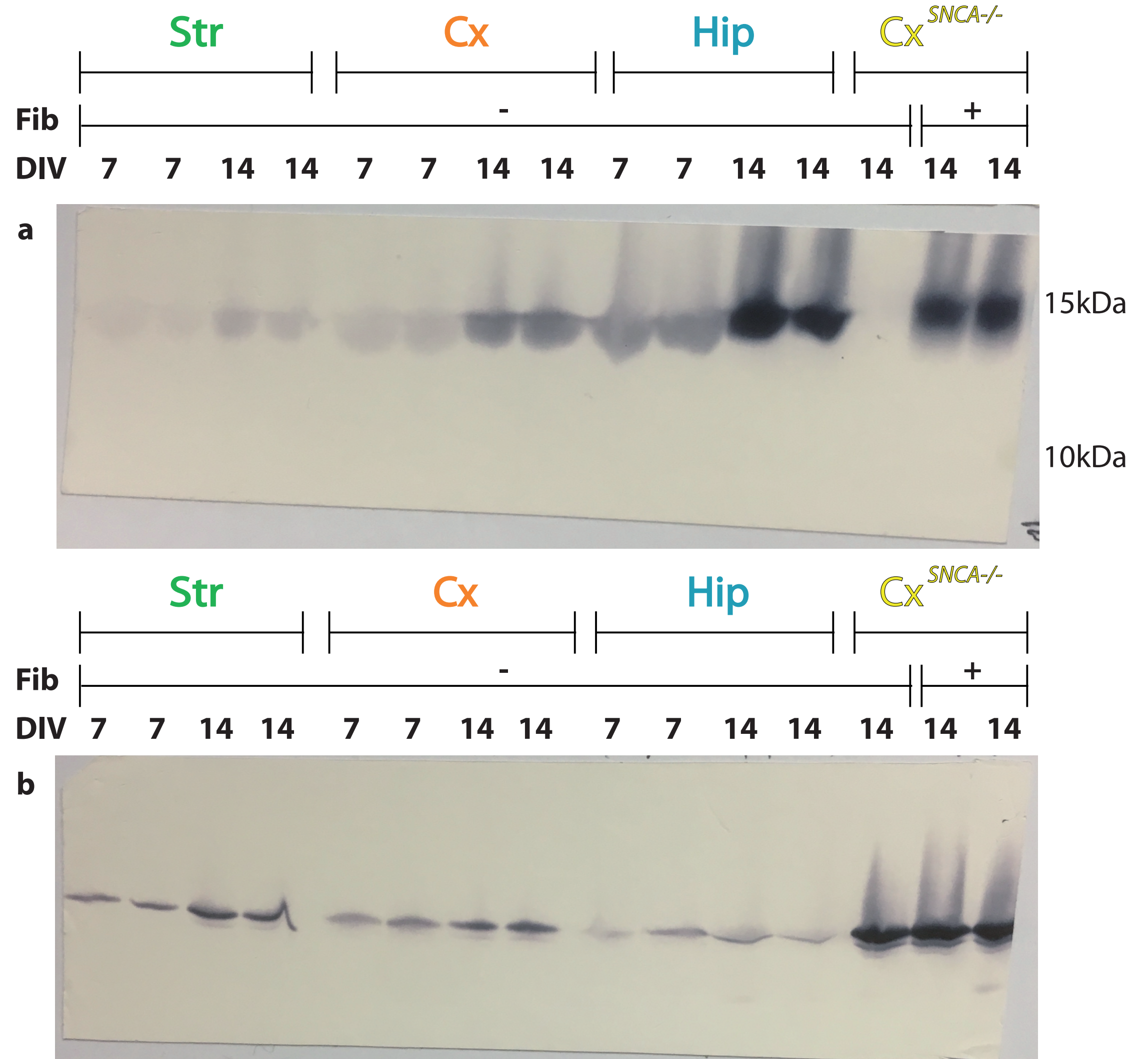
Str
Cx
Hip
Cx SNCA-/-
Fib
-
+
DIV 7 7 14 14 7 7 14 14 7 7 14 14 14 14 14
a
15kDa
10kDa
Str
Cx
Hip
Cx SNCA-/-
Fib
-
+
DIV 7 7 14 14 7 7 14 14 7 7 14 14 14 14 14
b

Supplementary Figure S11

Full-length gels for Figure 5b.

Primary neuronal cultures were lysed at DIV7 or DIV14, and lysates were submitted to gel electrophoresis. Transfer membranes were revealed with 2 different antibodies targeting aSyn **(a)** D37A6 from Cell Signaling Technology and **(b) and (d)** C20R from Abcam. Only (b) was kept for the main figure. **(c) and (e)** Transfer membrane revealed with TUJ1. Molecular weights markers are partially cropped but visible on the rightmost side of (a) and (b), and on the centre of (c). (a) is paired with the left part of (c), (b) with the right part of (c), and (d) with (e).

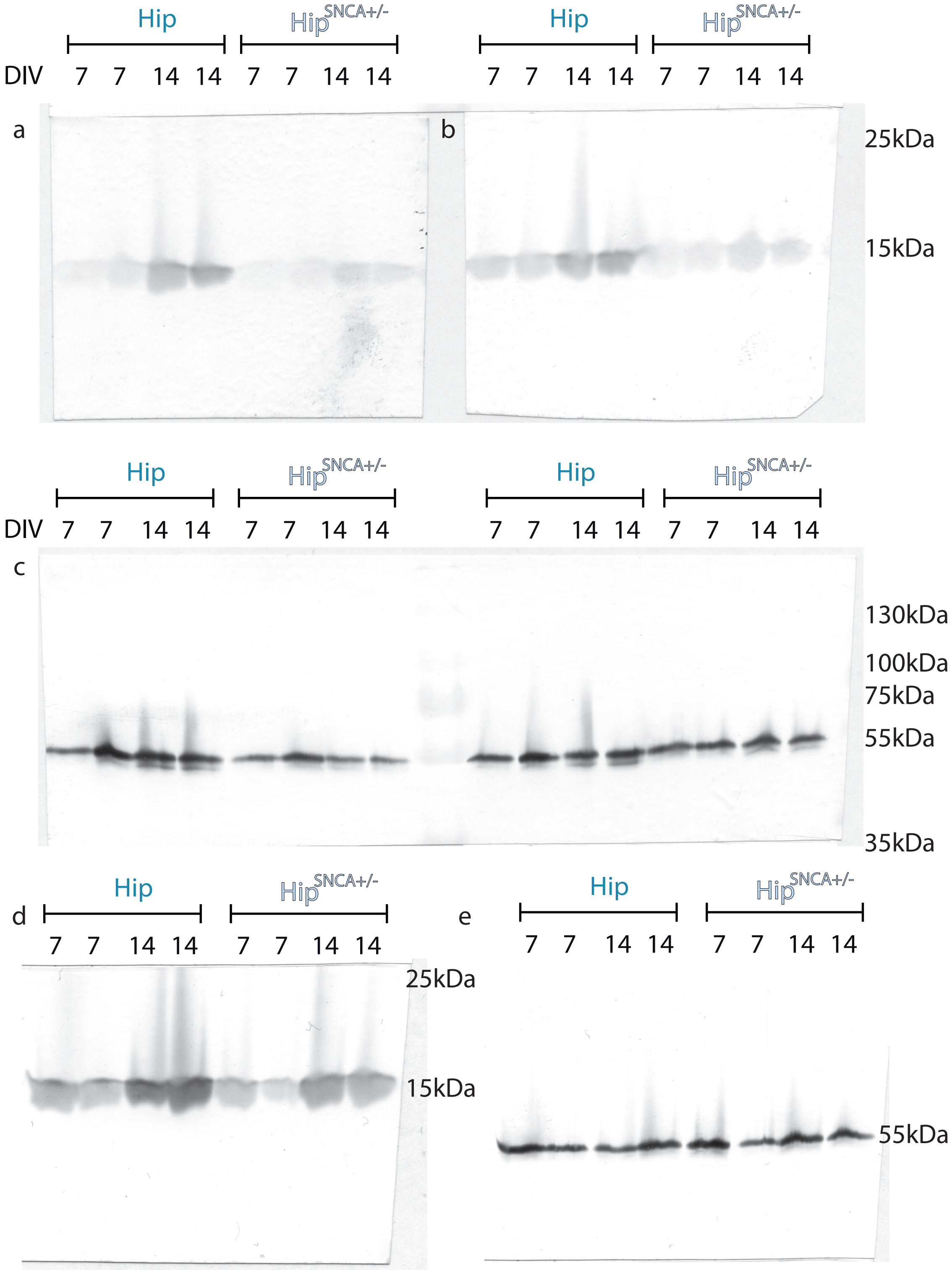
a
b
c
d
e
Hip
HipSNCA+/-
DIV
7 7 14 14 7 7 14 14
25kDa
15kDa
130kDa
100kDa
75kDa
55kDa
35kDa
